# The use of quetiapine in treatment of acute psychotic symptoms in an adolescent patient with primary brain calcification: a case report

**DOI:** 10.1186/s12888-019-2047-1

**Published:** 2019-02-11

**Authors:** Barbara Plemeniti Tololeski, Maruša Debeljak, Mirjana Perkovič Benedik, Tristan Rigler, Marinos Kyriakopoulos, Primož Kotnik, Katarina Šurlan Popovič, Maja Drobnič Radobuljac

**Affiliations:** 1 Centre for Mental Health, Unit for Adolescent Psychiatry, University Psychiatric Hospital Ljubljana, Grablovičeva 44a, 1000 Ljubljana, Slovenia; 2 Unit for Special Laboratory Diagnostics, University Medical Centre Ljubljana, University Children’s Hospital, Vrazov trg 2, 1000 Ljubljana, Slovenia; 3 Department of Pediatric Neurology, University Medical Centre Ljubljana, University Children’s Hospital, Bohoričeva ulica 20, 1000 Ljubljana, Slovenia; 4 South London and Maudsley NHS Foundation Trust, National and Specialist Inpatient Children’s Unit (Acorn Lodge), Tyson East Two, Bethlem Royal Hospital, Monks Orchard Road, London, BR3 3BX UK; 5 King’s College London, Department of Child and Adolescent Psychiatry, Institute of Psychiatry, Psychology and Neuroscience, 16 De Crespigny Park, London, SE5 8AF UK; 6 Medical Faculty, Chair of Pediatrics, University of Ljubljana, Bohoričeva ulica 20, 1000 Ljubljana, Slovenia; 7 Department of Pediatric Endocrinology, Diabetes and Metabolic Diseases, University Medical Centre Ljubljana, University Children’s Hospital, Bohoričeva ulica 20, 1000 Ljubljana, Slovenia; 8 Institute of Radiology, University Medical Centre Ljubljana, Zaloška cesta 7, 1000 Ljubljana, Slovenia; 9 Medical Faculty, Chair of Psychiatry, University of Ljubljana, Grablovičeva 44a, 1000 Ljubljana, Slovenia

**Keywords:** Primary brain calcification, Acute psychosis, Adolescent, Quetiapine

## Abstract

**Background:**

Primary brain calcification (PBC), a neurodegenerative disorder with characteristic calcium deposits in the basal ganglia and other brain areas, typically presents with various neurological and psychiatric symptoms in the fourth or fifth decade of life or later. We present the case of a patient with psychiatric manifestations much earlier than usual, in the second decade of life.

**Case presentation:**

The case of an adolescent female with acute psychotic symptoms, emotional instability, disorganized and suicidal behavior, stereotypical movements, below average intelligence and a three-year history of headaches is reported. Among others, the presentation included tactile hallucinations with secondary hypochondriacal delusions, which are rarely described in this diagnosis. Massive calcinations in the area of the basal ganglia and thalamus were determined by computerized tomography. Other causes of brain calcification were excluded. No causative mutations were found in selected genes. All the symptoms apart from lower intellectual abilities improved with quetiapine and sertraline. The patient showed no side effects.

**Conclusions:**

This case report highlights the successful use of quetiapine for symptomatic treatment of acute psychosis due to PBC in an adolescent without exacerbating extrapyramidal symptoms.

## Background

Primary brain calcification (PBC) is a neurodegenerative disorder with characteristic calcium deposits in the basal ganglia and other brain areas, visualized by neuroimaging, without defined cause (formerly known as idiopathic brain calcification) and also without the autosomal dominant pattern of inheritance seen in primary familial brain calcification (also known as Fahr’s disease) [1]. Most affected individuals are in good health during childhood and young adulthood and typically present in the fourth to fifth decade with gradually progressive neuropsychiatric and movement disorders. Neuropsychiatric symptoms, often the first or most prominent manifestation, range from mild difficulty with concentration and memory, to changes in personality and/or behavior, psychosis and dementia. Seizures of various types frequently occur, some individuals experience chronic headache and vertigo [2]. Computerized tomography is superior to magnetic resonance imaging for radiological diagnosis. No correlation has been identified between age of onset, extent of calcium deposits, and neurological deficits [3]. Treatment is currently symptomatic, aimed at improving the presenting neuropsychiatric symptoms, with a special warning for the cautious use of neuroleptic medications as they may exacerbate extrapyramidal symptoms [2].

## Case presentation

A 17-year-old female was admitted to the hospital due to severe suicidality. At the time of admission she complained about an irritating feeling in her nose, which made her constantly grimace in the area around the nose. She was excessively worried about having a serious illness of her nose (secondary hypochondriacal delusions) and was suicidal as a consequence. Her belief persisted even after any underlying medical condition of the nose has been ruled out by extensive medical examinations. She also presented with disorganized behavior, stereotypical movements, emotional instability and lability, and a below average level of intelligence during hospitalization. On the PANSS, her symptoms scored 29/23/70 (for the Psychotic, Negative and General Psychopathology Scale, respectively). Brief neurological examination revealed no abnormal neurological signs. As ascertained by the history taken from the patient and her mother, she had a history of school phobia that began at the age of 12 years, emotional disorders, normal cognitive and physical development, and a three-year history of chronic headache. She managed to complete primary and secondary education with the help of school counseling services given to her on account of school phobia. She had not received any psychiatric care before the described admission. A diagnostic evaluation for chronic headache at the University Children’s Hospital was undertaken a year before admission.

Calcium, phosphate and parathyroid hormone blood levels were normal. Vitamin D levels were decreased with decreased calcium levels in the urine. No signs of calcium depositions in organs other than the described brain regions were determined by ultrasound. Ophthalmological, ear-nose-and-throat examination and electroencephalography were also normal.

Detailed neurological examination revealed dysfunction of pursuit eye movement, dystonic positioning of both arms when stretched ahead, discrete ataxia of the arms and legs, and a pathological extensor response of the left big toe.

**Fig. 1:**
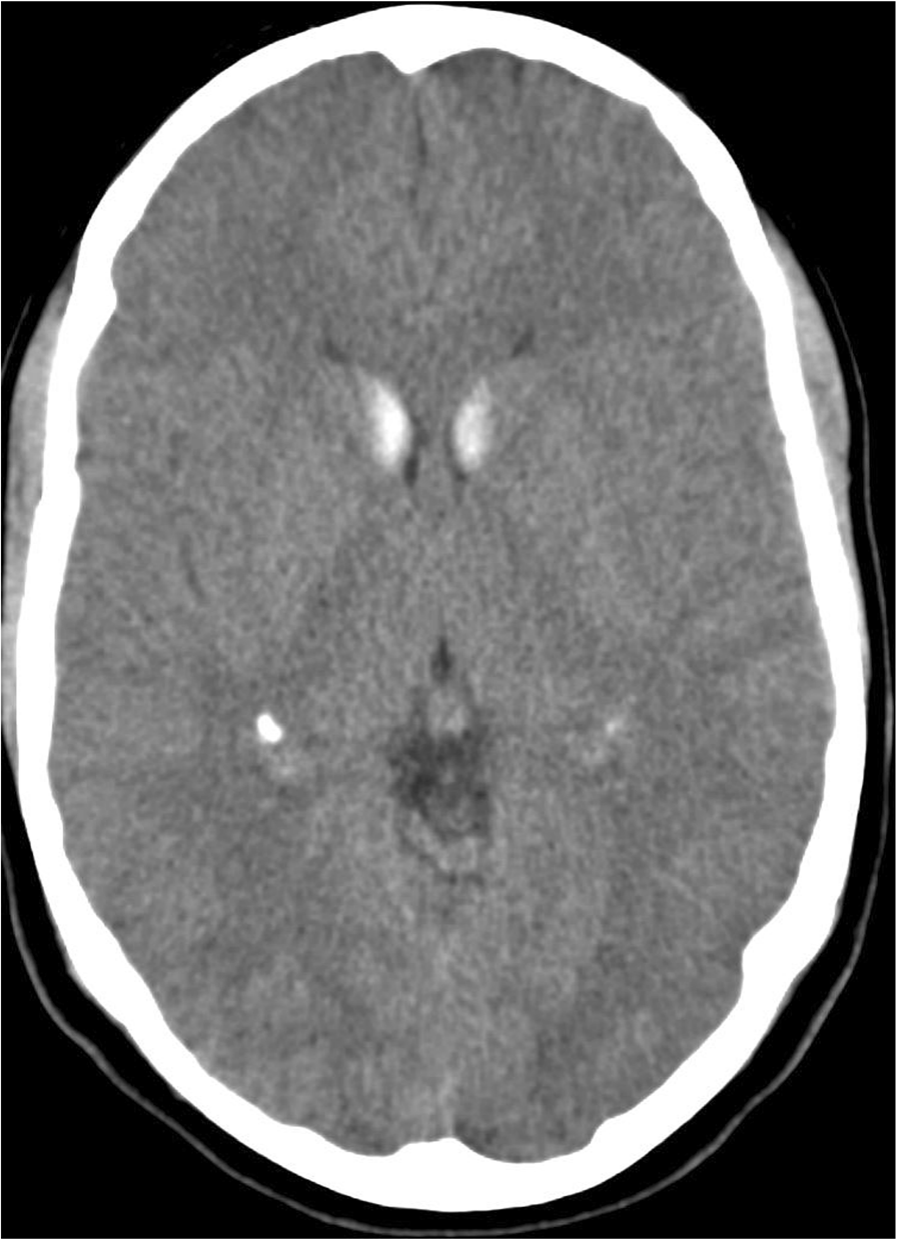
Computed tomography section of the patient showing bilateral symmetrical conflated calcifications involving the head of the caudate nucleus and thalamus

**Fig. 2:**
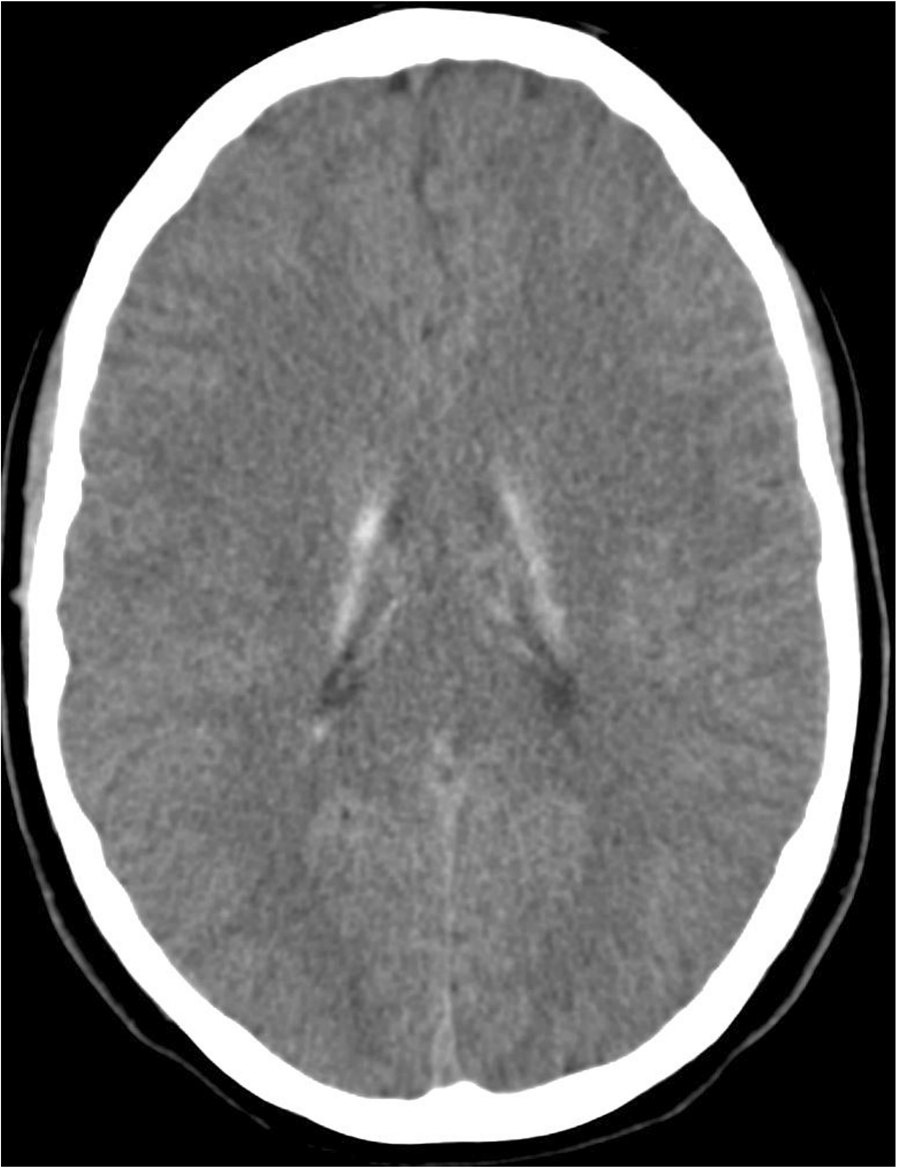
Computed tomography section of the patient showing bilateral symmetrical conflated calcifications involving the tail of the caudate nucleus

**Fig. 3:**
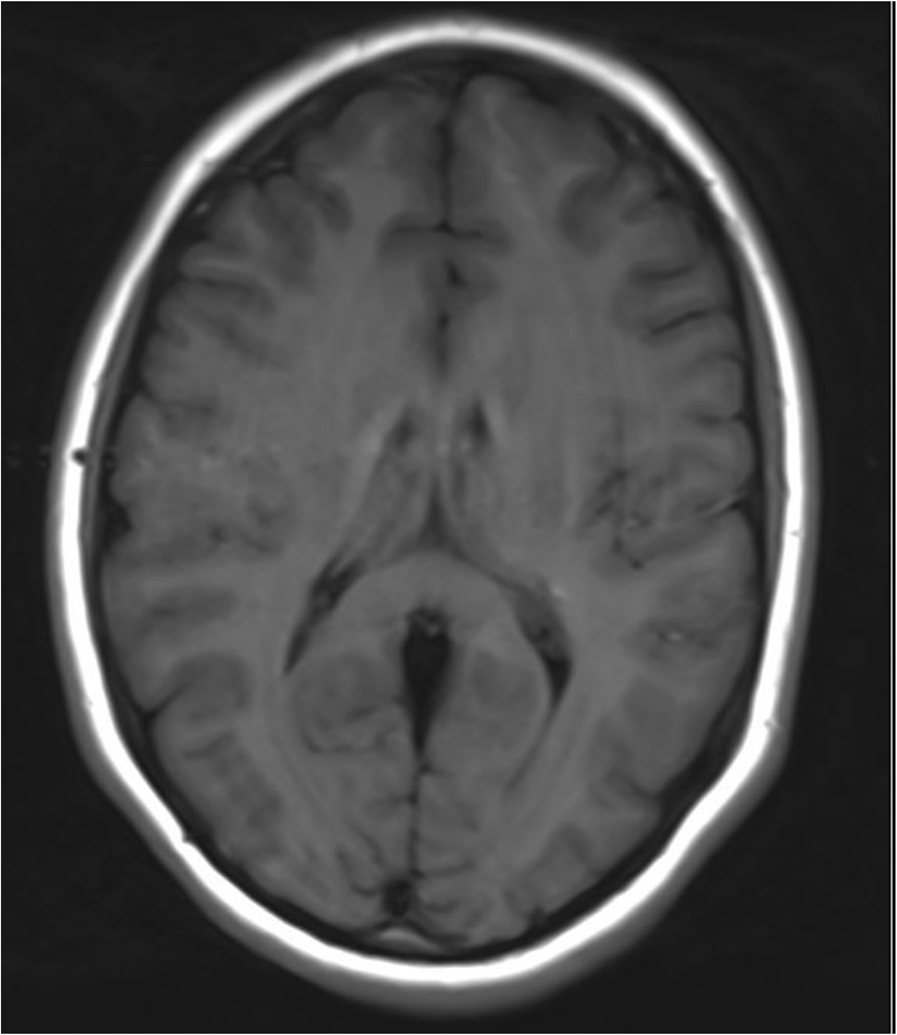
Corresponding MR T1 sequences show hyperintense calcifications in the same regions as those seen on CT examination

**Fig. 4:**
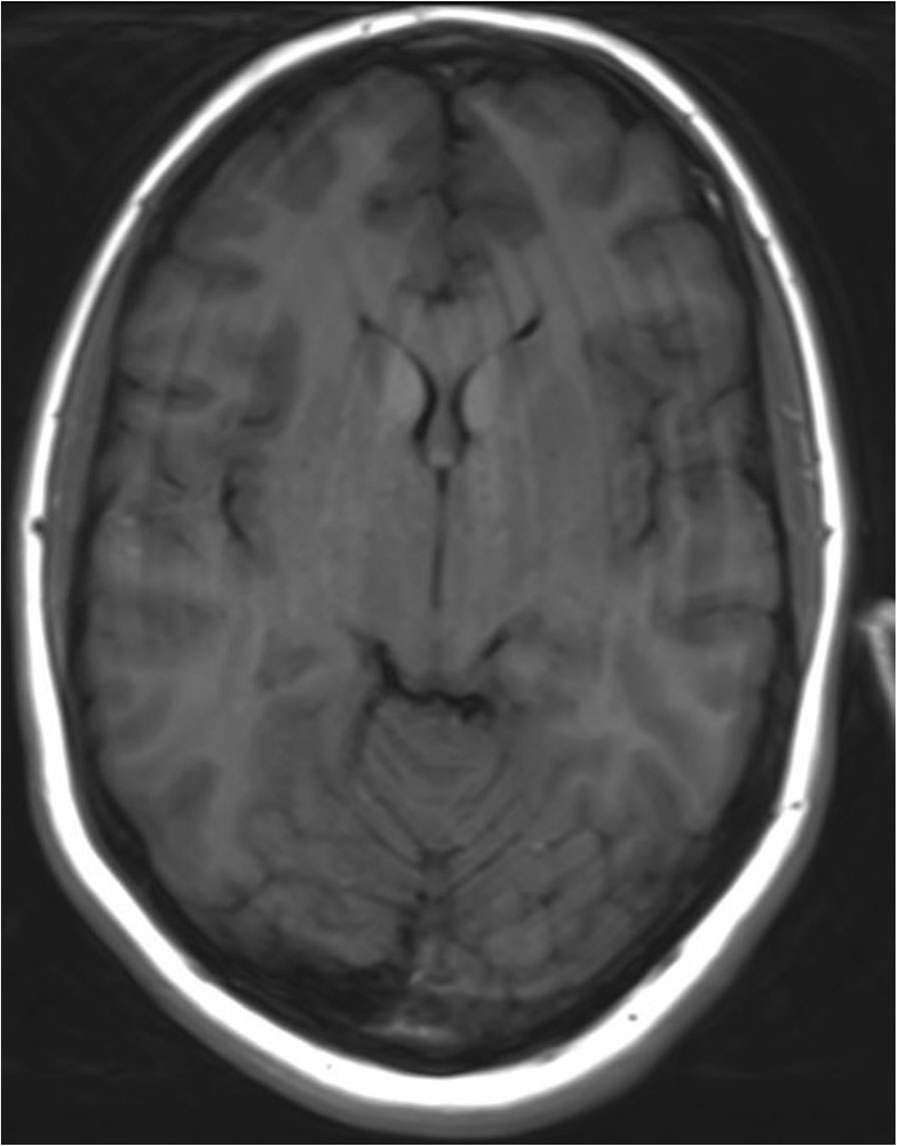
Corresponding MR T1 sequences show hyperintense calcifications in the same regions as those seen on CT examination

Bilateral symmetrical calcification in head, body and tail of the caudate nucleus and ventral part of the thalamus were determined by computerized tomography (Figs. 1 and 2). MRI was preformed twice over a two-year period using the same protocol. Corresponding MR T1 sequences showed hyperintense calcifications in the same regions as those found on CT examination (Figs. 3 and 4). Both MR examinations showed a high signal of calcified areas on T1 weighted sequences due to the surface area of calcium crystals. The same areas had an isointense signal on T2 weighted sequences. No other abnormalities of the brain were detected on the MRI.

During psychometric evaluation, the patient’s cognitive abilities were assessed with RPM, TOL-II, d2, CTMT and Stroop tests. The patient performed significantly worse than her normative age group in terms of general cognitive abilities, coming in below the 10th percentile. She was unable to perform problem-solving operations that require abstract thinking. Assessment of her attention performance showed below-average results in scanning and alternating attention. She also showed below-average performance in her sustained and divided attention, with her concentration performance in the 8th percentile. Her planning abilities were significantly worse in comparison with her normative group, where she was unable to construct a problem-solving strategy. Her approach was a trial-and-error strategy and she failed to solve the problem within the time limit. The patient had no significant difficulties with inhibition of dominant response, reaching a borderline average result. We performed a retest after a year of treatment and the results showed no significant changes, although she was slightly better, but unfortunately without significant improvements, in her planning abilities and in her sustained attention.

A whole blood EDTA sample was used for extraction of genomic DNA according to established laboratory protocols using the FlexiGene DNA isolation kit (Qiagen, Germany). Whole exome sequencing *a trio* (index patient and her parents) was performed in collaboration with NovoGene Corp. Inc. (Davis, CA, USA) using an Agilent Sure Select Human All Exon V6, 5191–4004 kit for whole exome enrichment preparation together with an Illumina Platform PE150 (Illumina, San Diego, USA) to perform the whole exome sequencing. Genetic variants with coverage >15x were analyzed using Variant Studio 3.0 software (Illumina). Evaluation of variants was firstly restricted to those located in eight genes related to Fahr’s disease (*SLC20A2, PDGFRB, PDGFB, XPR1, KRIT1, SLC19A3, TREX1, MYORG*). We reached 99.9% with at least 10X coverage for the patient. A search tool for the retrieval of interacting genes/proteins (STRING, http://string-db.org/) was used to construct the protein-protein interactions that are involved downstream and upstream in Fahr’s syndrome (*GNAS, ERCC8, PDGFB, CYP2U1, GNA11, SLC20A2, IFIH1, PSMB8, PDGFRB, CA2, ERCC6, SAMHD1, TREX1, CASR, TREM2, TYROBP, GJA1, ERCC3, FAM111A, RNASEH2B, SLC46A1, SLC7A7, ATP13A2, PARK7, HMBS, KRIT1).* No causative mutations were found in the selected genes in the patient.

Computerized tomography scans of the heads of the patient’s parents were normal.

The patient was treated symptomatically with quetiapine sustained release (initially 200 mg and gradually increasing to 900 mg daily) and sertraline (150 mg daily, gradually increasing to 200 mg daily). We did not observe any side effects with the use of quetiapine, although special attention was given to the possible exacerbation of extrapyramidal symptoms. Psychotic (PANSS scores at discharge were 8/13/27, PANSS scores after two years were 9/16/34 for the Psychotic, Negative and General Psychopathology Scale, respectively), affective and behavioral symptoms were improved; she was no longer suicidal and remained stable on gradually increasing doses of antipsychotic medication within two years of treatment, however, her intellectual abilities were not improved. Even though the patient completed secondary professional education and intense professional rehabilitation efforts were made, she has not been able to start working, mainly due to emotional instability. The patient was transferred to adult psychiatric services at the age of 21.

## Discussion and conclusions

The clinical expression of PBC can vary greatly. Symptoms can include psychiatric disorders, neurological symptoms and even significant cognitive deterioration such as dementia [4]. The most common psychiatric manifestations are mood disorders. Psychotic symptoms usually include auditory and visual hallucinations, complex perceptual distortions, delusions and fugue states [5]. Most affected individuals are in good health during childhood and young adulthood [2]. In our case, symptoms started much earlier than usual, as a seventeen-year-old, and presented with psychotic rather than affective symptoms.

Even though there are other cases of affected adolescents found in the literature, for example an eighteen-year-old with manic symptoms [6] and a seventeen-year-old with catatonia associated with comorbid bipolar affective disorder [7], our case presented primarily with psychotic symptoms at a similar age. This shows that the disease may not be as rare in adolescence as previously believed and is becoming more frequently diagnosed due to the increased use of neuroimaging even in younger patients. Therefore psychiatrists should consider PBC as a differential diagnosis in the evaluation of psychosis and comorbid mood disorders associated with movement abnormalities, even as early as adolescence.

Our case presented with unusual psychotic symptoms including tactile hallucinations in the nasal mucosa and secondary delusions. Similar psychopathology (tactile hallucinations in the chest and paranoid delusions) was described in the case of a 60-year old female with possible PBC [8]. It is possible that tactile hallucinations are not that uncommon in PBC and represent an important part of a varied clinical picture of atypical psychosis, even in younger patients with PBC, as shown in our case.

When a diagnosis of PBC is considered, clinical, laboratory and radiologic investigations in first-degree relatives should also be performed. Although several genes are already associated with the development of the disease, it is possible that in the future others may be determined, further illuminating the pathophysiological mechanisms of this puzzling condition.

There is currently no cure for PBC or even a standard course of treatment. The available treatment is directed towards control of symptoms. However, when using antipsychotics, it is important to take into consideration that patients with PBC are more susceptible to neuroleptic malignant syndrome when treated with antipsychotic drugs [5]. Cautious use of antipsychotic medication is also important as it may exacerbate extrapyramidal symptoms [9]. Therefore, we have chosen an atypical antipsychotic quetiapine, since data in the literature claimed that it was associated with a placebo-level incidence of extrapyramidal symptoms (EPS) across its entire dose range, appeared to have a low risk for EPS in vulnerable patient groups (e.g. the elderly, adolescents or patients with organic brain disorders) and had a more favourable EPS profile than risperidone [10]. Quetiapine has been successfully used in other patients with PBC without causing EPS, according to a few case reports [7, 11]. In our case the use of quetiapine resulted in good remission of psychotic symptoms and suicidality without causing any neurological deterioration.

A decline in cognitive function was a very important symptom in the patient while she has not shown any signs of significantly impaired premorbid intellectual functioning. Additionally, there was no improvement in cognitive abilities after remission of the acute psychosis. Due to the short period of observation and known instability of clinical presentation in this condition, it is hard to predict the course of progression in this case. Nevertheless, we fear that the patient may progressively deteriorate and require more support in terms of psychoactive medications for the treatment of psychotic symptoms and affective instability, as the need for gradually increasing doses of medications required so far indicates. Another complicating factor is her inability to work, which suggests that the patient will be unable to care for herself unaided. It is important to consider PBC as a differential diagnosis in adolescent patients, because with more successful treatment of symptoms these patients may still be able to undertake more education and achieve greater autonomy in adulthood.

## Abbreviations

CTMT: Comprehensive Trail-Making Test
DNA: Deoxyribonucleic acid
EDTA: Ethylenediaminetetraacetic acid
MRI: Magnetic Resonance Imaging
PANSS: Positive and Negative Syndrome Scale
RPM: Raven’s Progressive Matrices
TOL-II: Tower of London Test
